# Penetration of an antimicrobial zinc-sugar alcohol complex into *Streptococcus mutans* biofilms

**DOI:** 10.1038/s41598-018-34366-y

**Published:** 2018-11-01

**Authors:** Jong Hyun Lim, Yongbeom Jeong, Sang-Hun Song, Jae-Hyun Ahn, Jeong Rae Lee, Sang-Min Lee

**Affiliations:** Magok R&D Center, LG Household & Health Care, Gangseo-gu, Seoul, 07795 Republic of Korea

## Abstract

Mature biofilms are highly resistant to antimicrobial agents due to the presence of extracellular polymeric substances (EPS), which inhibit the penetration of external molecules. In this study, we developed a coordination compound consisting of zinc chloride and erythritol that exhibits penetrating and bactericidal activity against *Streptococcus mutans* biofilms. An *in vitro* biofilm model was established in microplates, and bactericidal activity against biofilms was evaluated using an Alamar blue assay. The cause of the antimicrobial activity of the zinc-erythritol mixture on mature biofilms was demonstrated using fast atom bombardment-mass spectrometry, confocal laser scanning microscopy and atomic force microscopy. We demonstrated that zinc chloride spontaneously formed cationic complexes with erythritol in water. The zinc-erythritol complexes reduced intra- and inter-molecular interactions between bacterial exopolysaccharides, a major component of EPS. This activity was confirmed by measuring the attenuation of the hardness of dried polysaccharides isolated from *S. mutans* biofilms. The reduction in the interactions between polysaccharides allowed the complexes to penetrate into biofilms and kill the embedded bacteria. While approximately 13% of biofilm-associated microbes were killed by a 10 min treatment with 6.6 mM zinc chloride, 45% were killed when a solution containing 19.8 mM erythritol and 6.6 mM zinc chloride was used. This strategy of leveraging the coordination properties of metal ions with sugar alcohols provides a simple way to effectively remove mature biofilms using only conventional substances without the need for intricate chemical synthesis processes.

## Introduction

Biofilms are microbial communities of bacterial cells that are firmly attached surfaces and are surrounded by a protective three-dimensional matrix of extracellular polymeric substances (EPS). This structure provides an environment that helps microorganisms exhibit excellent resistance to antimicrobial compounds^1^. Microbes in biofilms can be as much as 1,000 times more resistant to antimicrobial agents than planktonic ones^2^, making biofilms difficult to eradicate. In particular, the removal of biofilms in the human body requires that many factors be considered, such as safety and short treatment time. Among the human body, to remove biofilms in the oral cavity, only extremely limited substances are available, because their absorption into mucosal tissues and ingestion must be additionally considered^3,4^.

Oral biofilms are widely known to cause periodontal diseases, such as gingivitis, periodontitis and dental caries^5,6^. To remove oral biofilms, a variety of studies have been conducted using enzymes or novel substances. Enzymes can effectively degrade biofilms under mild conditions^7,8^, but they must overcome major drawbacks, such as instability and long reaction times. Biofilms can also be exfoliated when losing their adhesive force by dissolution of EPS^9^. Nonetheless, this method has the limitation that the biofilm is not completely eradicated and can form again within 2–4 hours^10,11^. Thus, research into the development of antimicrobial agents that can eradicate bacteria in mature biofilms is required for their more long-term removal.

Many researchers have proposed newly-synthesized antimicrobial materials with excellent biofilm-penetrating abilities, such as antimicrobial peptides and nanoparticles^12–15^. These novel materials have unique characteristics that react to pH^13^ or penetrate biofilms by magnetic forces^15^ to enhance their antibacterial effect on biofilms. However, most of these materials cannot be actually utilized due to their potential toxicity. Therefore, for practical purposes, it is desirable to only use substances that have been fully demonstrated to be safe for human health and that can be exposed to the oral environment. Zinc and sugar alcohols are potentially preferred candidate materials for removing biofilms. Although large amounts of zinc ions are potentially toxic^16^, small amounts are essential for metabolic processes^17^ and have a positive impact on bone formation^18,19^. In addition, zinc ions act as an antimicrobial agent by deactivating proteins, causing structural changes in microbial membranes and affecting microbial nucleic acids^20–22^, although the efficacy is insufficient to eradicate mature biofilms. Sugar alcohols such as xylitol are some of the most widely used substances to control oral biofilms due to their safety to human health and their ability to inhibit the formation of biofilms^23^. In particular, xylitol-containing chewing gums are capable of inhibiting the formation of oral biofilms due to their ability to stimulate salivation^24,25^, and they are widely used worldwide.

Erythritol is a 4-carbon sugar alcohol that has recently attracted great attention for its superior ability to inhibit the formation of oral biofilms compared to xylitol^26^. In addition, erythritol has been reported to enhance the penetration of antimicrobial agents such as benzethonium chloride and chlorhexidine into biofilms^27,28^. This penetration-enhancing effect was speculated to be caused by the diffusion of erythritol into biofilms^27^, but the exact mechanism is unclear. And its applicability in various situations is unfortunately limited, because the effect only occurs at high concentrations (>5% by weight). In this study, we hypothesized that the biofilm-disrupting efficacies of zinc and erythritol could be synergistically enhanced by the formation of complexes. To confirm this hypothesis, a strategy to form cationic complexes by coordination bonds between zinc chloride and alcohols was adopted. The cationic complexes were delivered to negatively-charged biofilms and penetrated into *Streptococcus mutans* biofilms by weakening the cohesion of exopolysaccharides in biofilms. The results of this study demonstrate the antimicrobial effect of the zinc-erythritol complexes on mature biofilms, and an interpretation of the cause of their penetration is present.

## Results

### Antimicrobial effect of zinc chloride-erythritol mixture against *S. mutans* biofilm

**Figure 1:**
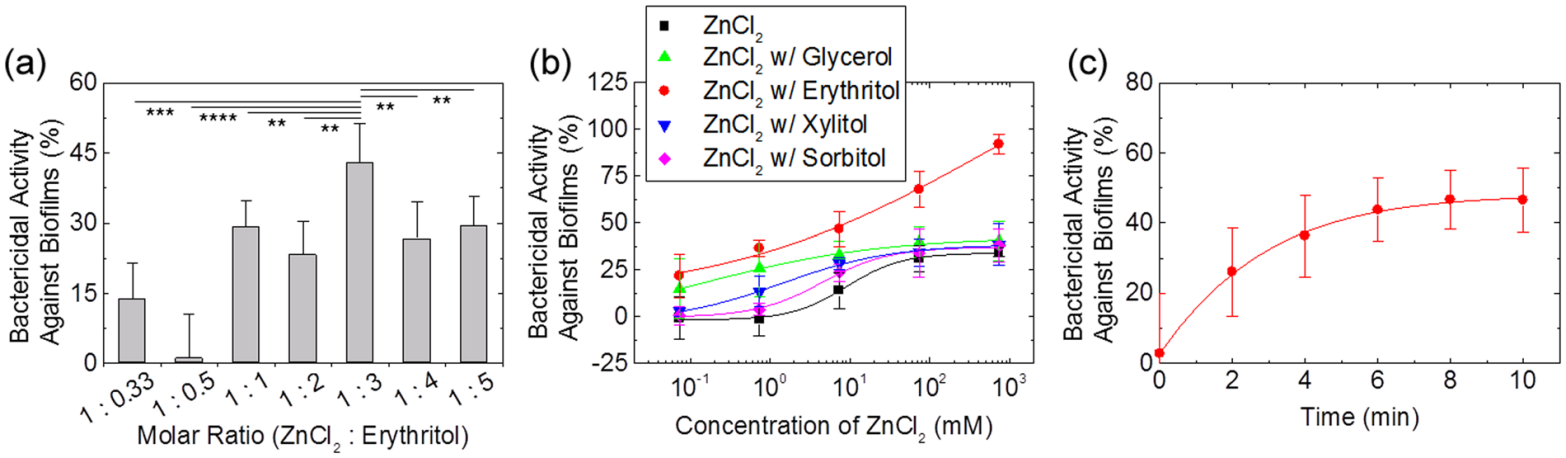
Bactericidal activity of mixtures of zinc chloride and erythritol against *S. mutans* biofilms. (**a**) Effect of the molar ratio used for zinc chloride and erythritol on antibiofilm activity. The concentration of zinc chloride used was 6.6 mM, and the concentration of erythritol varied according to the indicated ratio. Significance was calculated using a Student’s t-test. (**b**) The effect of ligands combined with zinc chloride on the antimicrobial activity against biofilms. The concentration of zinc chloride was 6.6 mM. (**c**) Time-dependent correlation between antibiofilm activity and exposure time of the solution containing zinc chloride and erythritol at a molar ratio of 1:3. In this experiment, the solution containing 6.6 mM zinc chloride and 19.8 mM erythritol was used. In all figures, each value and error bar indicates the mean and standard deviation (SD), respectively (*n* = 5–10).

Zinc chloride has no significant effect on mature biofilms with antimicrobial resistance when used for short exposure times. However, we demonstrated that bacteria embedded in biofilms can be killed when treated with a mixture of zinc chloride and erythritol at a specific ratio. As shown in Fig. 1a, a mixture of zinc chloride and erythritol at a molar ratio of 1:3 exhibited the highest biofilm-removal efficiency. In addition, the effect tended to decrease as the deviation from the optimal ratio increased.

The types of sugar alcohols paired with zinc chloride influenced the observed bactericidal activity against biofilms. Aqueous solutions containing ZnCl_2_-glycerol, -erythritol, -xylitol, and -sorbitol were tested. The molar ratio between ZnCl_2_ and sugar alcohols was chosen to be the most efficient for the bactericidal activity against *S. mutans* biofilms. For ZnCl_2_-glycerol, the bactericidal activity against biofilms was the strongest at a ratio of 1:3, whereas the optimum ratio of ZnCl_2_-xylitol and ZnCl_2_-sorbitol was 1:4 (Fig. S1). And, as shown in Fig. S2, a short-term exposure for 10 min of sugar alcohols had no effect on reducing bacterial viability in mature biofilms. However, glycerol and erythritol significantly improved the antimicrobial effect of zinc chloride against biofilms (Fig. 1b). In particular, erythritol caused a remarkable increase in antimicrobial activity at concentrations above 6.6 mM.

The time required to remove biofilms is an important factor to be considered, especially in the case of oral biofilms, where a short treatment time is highly desirable. *S. mutans* biofilms were immersed in a solution containing zinc chloride and erythritol for different periods of time. As shown in Fig. 1c, the biofilm-removal efficiency increased with contact time, plateauing at approximately 5 min. More than 70% of the effect was achieved in approximately 3 min.

The efficacy of the zinc-erythritol mixture was affected by the pH of solutions because zinc hydroxide was formed at high pH values. As expected, the antimicrobial activities of zinc chloride and the zinc-erythritol mixture against biofilms were weakened as the pH of the solution increased (Fig. S3). Nonetheless, regardless of pH, the tendency for significantly increased antibiofilm activity when zinc was combined with erythritol was consistent.

### Penetration of zinc and erythritol into biofilms

Further studies have been conducted to elucidate the mechanism of the activity of the zinc-erythritol mixture against *S. mutans* biofilms. First, we tested whether erythritol enhances the bactericidal activity of zinc chloride against planktonic microbes. As shown in Fig. S4, when the incubation time was 0 h (i.e., before biofilms were formed), the ability of the zinc-erythritol mixture to kill bacteria was not superior to zinc chloride itself. This result shows the percentage of killed planktonic microorganisms when contacted with the zinc chloride or zinc chloride-erythritol mixture for 10 min prior to biofilm formation. Thus, the bactericidal activity of zinc chloride against planktonic microbes was not enhanced by the combination with erythritol.

However, after the biofilms had formed, zinc chloride without erythritol rapidly lost its antimicrobial activity (Fig. S4). The effect of the zinc-erythritol mixture also decreased with maturation of the biofilm, but the rate of decrease was much slower than that of zinc chloride. Considering that the bactericidal activity of the zinc-erythritol mixture was less affected by the maturity of the biofilm than zinc chloride, we deduced that zinc chloride and erythritol penetrated the biofilm.

**Figure 2:**
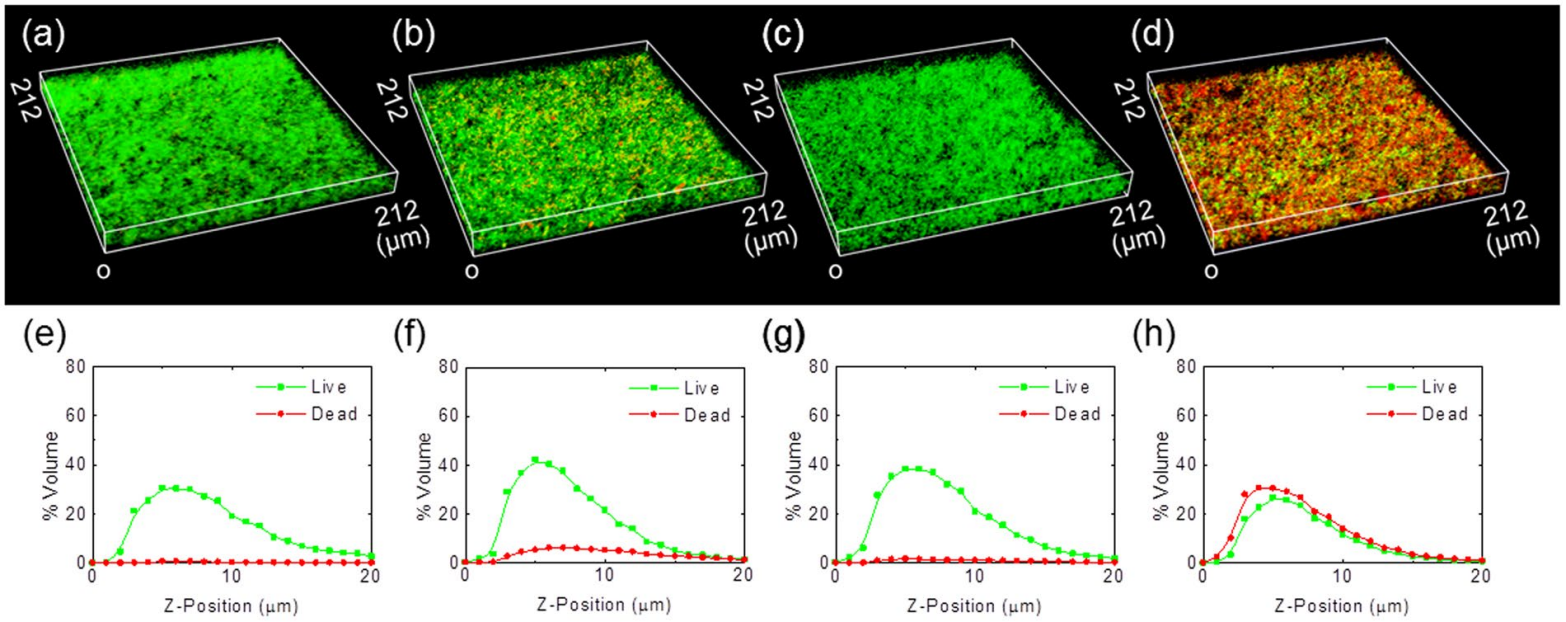
CLSM images of *S. mutans* biofilms after treatment with zinc chloride and erythritol. Live (green) and dead (red) bacteria were imaged using CLSM (**a–d**). Biofilms treated with (**a**) DW, (**b**) zinc chloride, (**c**) erythritol and (**d**) a zinc-erythritol mixture for 10 min. The concentrations of zinc chloride and erythritol were 6.6 and 19.8 mM, respectively. The percentages of the volume occupied by live (green) and dead (red) bacteria was plotted (**e–h**). Data were quantified using image slices of z-stacks obtained from stained biofilms treated with (**e**) DW, (**f**) zinc chloride, (**g**) erythritol and (**h**) a zinc-erythritol mixture.

The penetration of the zinc-erythritol mixture into mature biofilms was more definitively shown by confocal laser scanning microscopy (CLSM). After the treatment with 6.6 mM zinc chloride and 19.8 mM erythritol, bacterial cells were stained with SYTO9^TM^ and propidium iodide (PI) to discriminate between live and dead cells. Live and dead cells are shown in Fig. 2 as green and red fluorescence, respectively. Although zinc chloride and erythritol were ineffective as antimicrobial agent against mature biofilms by themselves, a mixture of zinc chloride and erythritol penetrated deep into the biofilm and killed the embedded bacteria.

### Weakening of the hardness of exopolysaccharides in biofilms

**Figure 3:**
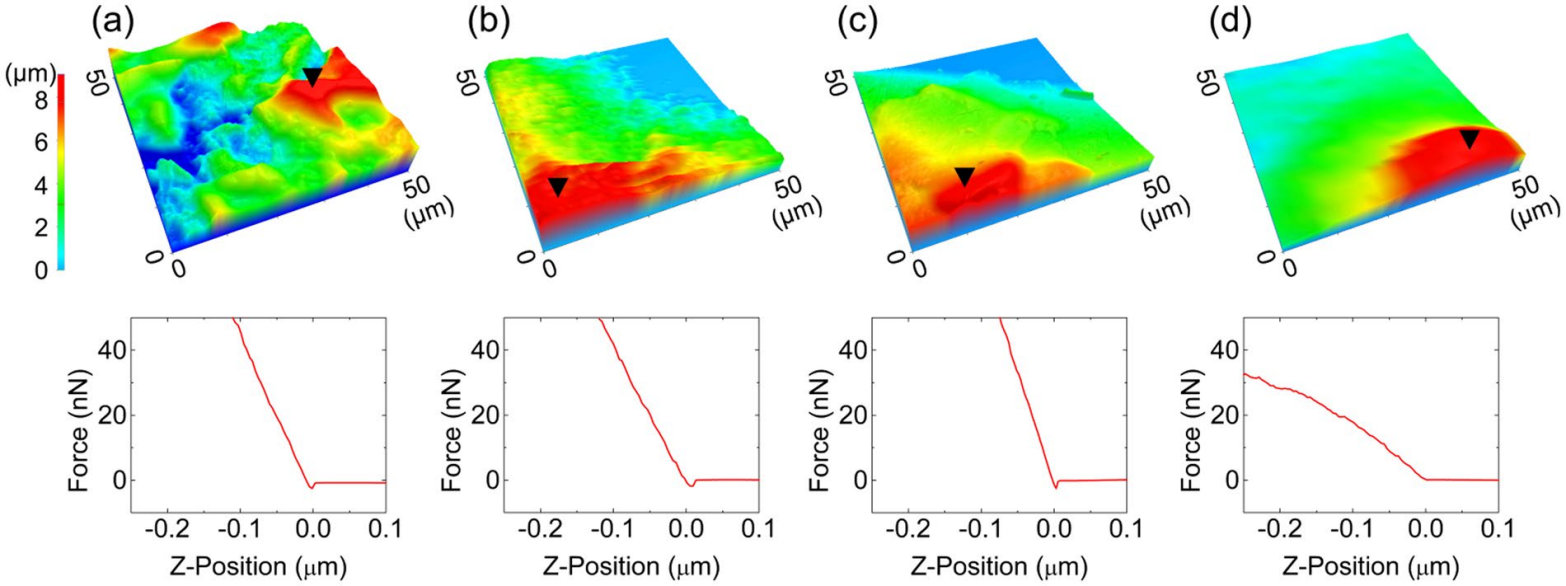
Analysis of the hardness of polysaccharide particles isolated from an *S. mutans* biofilm. AFM images (upper) of polysaccharide particle morphologies and force-distance curves (lower) after treatment with (**a**) DW, (**b**) zinc chloride, (**c**) erythritol and (**d**) a zinc-erythritol mixture for 10 min. The polysaccharide particles were treated with 6.6 mM zinc chloride, 19.8 mM erythritol or mixtures thereof. The inverted triangle represents where the force-distance curve was measured.

To determine the cause of the observed biofilm penetration by the zinc-erythritol mixture, we assessed the effect of the mixture on exopolysaccharides isolated from *S. mutans* biofilm, which are insoluble in water and exist as micro-sized particles when dried. The exopolysaccharides in *S. mutans* biofilms are densely aggregated by intra- and inter-molecular interactions to block the penetration of external substances. The effect of zinc chloride and erythritol on this aggregation was evaluated by measuring the hardness of the polysaccharide particles with an atomic force microscope (AFM). A repulsive force is generated when the AFM tip attempts to penetrate the particle by moving vertically. The repulsive force is expressed as a slope in the force-distance spectroscopy graph. The polysaccharides interfered with tip motion with constant force (i.e., a linear slope), as shown in Figs 3a and S5a. This property did not change after contact with zinc chloride or erythritol (Figs 3b,c, S5b and S5c). However, upon contact with a zinc-erythritol mixture, a unique pattern was observed when AFM tip was moved. As the tip penetrated, the repulsive force gradually decreased (Figs 3d and S5d).

### Effect of the zinc-erythritol mixture on human oral keratinocytes

**Figure 4:**
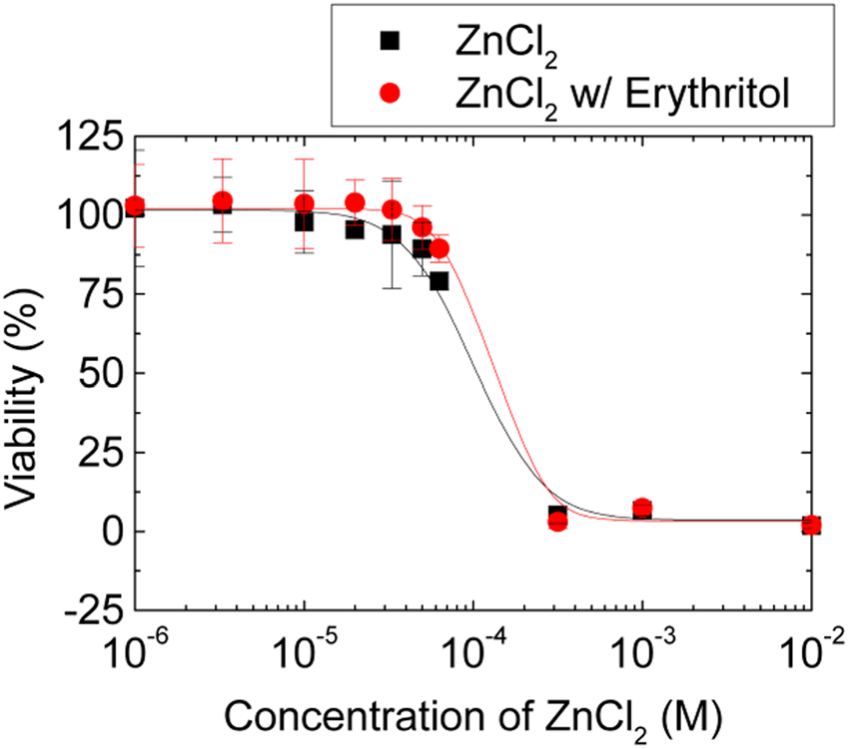
Cytotoxicity of zinc chloride and a zinc-erythritol mixture. The viability of human oral keratinocytes was determined after treatment with zinc chloride or a zinc-erythritol mixture. The zinc-erythritol mixture contained zinc chloride and erythritol at a molar ratio of 1:3. Each value and error bar indicates the mean and SD, respectively (*n* = 10).

The combination of zinc chloride and erythritol did not cause additional toxicological problems as confirmed by measuring the cytotoxicity of the mixture to human oral keratinocytes. Erythritol had no effect on cell viability by itself (Fig. S6), whereas zinc chloride has toxicological potential and had a negative impact on cells at high concentrations (>10^−4^ M) as shown in Fig. 4. The cytotoxicity of the zinc-erythritol mixture at high concentrations was similar to that of zinc chloride. However, when the concentration used was below 10^−4^ M, the mixture showed slightly lower cytotoxicity than zinc chloride. Thus, the half-maximal inhibitory concentration (IC50) value of the mixture was higher, although not significantly, than that of zinc chloride. The IC50 values of the mixture and of zinc chloride were 0.13 ± 0.02 and 0.10 ± 0.01 mM, respectively.

## Discussion

In this study, the concentration of zinc chloride used was fixed at 6.6 mM (0.09% by weight). Mouthrinses containing 6.6 mM zinc chloride have been clinically demonstrated to have an anti-calculus effect^29,30^, and most commercial oral care products on the world market, such as toothpastes and mouthrinses, actually include 6.6 mM zinc chloride as an active ingredient. In this context, a concentration of 6.6 mM zinc chloride is highly relevant. In addition, we plan to clinically validate whether erythritol has a positive impact on zinc chloride-containing oral care products. Therefore, the efficacy of zinc chloride at 6.6 mM was verified in this study.

As shown in Fig. 1a, the antibiofilm efficacy of the zinc-erythritol mixture appears only at a certain ratio, which is reminiscent of type IV eutectic phenomena between metal chlorides and donor molecules^31^. Deep eutectic solvents (DESs) have been reported that consist of zinc chloride and diol donors, such as ethylene glycol and 1,6-hexanediol^32^. Cationic complexes were observed to be formed in DESs when the molar ratio of zinc chloride/diol molecules was 1:3 or 1:4. In our study, a tetraol molecule, erythritol, was used instead of diols with zinc chloride. The mixture of zinc chloride and erythritol is not a eutectic mixture because it completely dissolved in water. Nevertheless, as confirmed by fast atom bombardment mass spectrometry (FAB-MS) analysis in Fig. S7, the intermolecular interactions between zinc chloride and erythritol in water appeared similar to those observed in DESs. Zinc chloride and erythritol presumably were connected by coordination bonds such that cationic complexes such as [ZnCl(erythritol)]^+^ and [ZnCl(erythritol)_2_]^+^ could be formed. A mixture of zinc chloride and erythritol at a molar ratio of 1:3 was fully dissolved in deionized water, and the solution was analyzed by FAB-MS. Thus, the spectrum shown in Fig. S7 may be evidence to demonstrate the possibility of the presence of cationic complexes composed of zinc chloride and erythritol even though they were dissolved in water.

More definitive evidence for the complexation between erythritol and zinc nitrate was previously obtained by Yang *et al*.^33^, who used Fourier-transform infrared spectroscopy and X-ray diffraction analysis to demonstrate that Zn^2+^ is coordinated to hydroxyl groups of erythritol to form a zinc nitrate-erythritol complex. The results of their study support our hypothesis that zinc salts are associated with erythritol through coordination bonds. However, unexpectedly, the presence of erythritol did not significantly affect the bactericidal activity of other zinc salts, such as zinc citrate, zinc sulfate and zinc nitrate against *S. mutans* biofilms, as shown in Fig. S8. This result is presumably due to a larger size of counter ions compared to chloride ions, which can limit diffusion of the complexes into the exopolysaccharides of biofilms. Or, more realistically, it may be because we could not identify specific conditions required for the complexation to occur.

The bactericidal activity of the zinc chloride-erythritol mixture comes from zinc chloride^20–22^. However, the ability of zinc chloride to penetrate the EPS of biofilms is an important factor in the antibiofilm activity. The results shown in Fig. S4 demonstrate that the bactericidal activity of zinc chloride lowers on biofilms than on planktonic cells, indicating that zinc did not penetrate the EPS of *S. mutans* biofilms. Furthermore, the addition of erythritol did not enhance the antimicrobial activity of ZnCl_2_ against planktonic bacteria (Fig. S4). However, when erythritol was added to the ZnCl_2_ solution, the bactericidal activity of the mixture against biofilms was less affected by the formation of biofilms. Therefore, we interpreted that this phenomenon might be due to an increase in the penetration of zinc against mature biofilms due to the presence of erythritol.

In the AFM experiments, we observed that the zinc-erythritol mixture reduced the repulsive force generated when the AFM tip penetrated the extracellular polysaccharides of biofilms. The reduction in repulsive force was presumably due to the physical breakage of the particles by the pressure of the tip. If the structure of the polysaccharide particles is destroyed by the penetration of the tip, the repulsive force will be weakened due to the decrease in obstacles hindering the movement of the tip. Because the destruction of the particles indicates that the internal cohesion of the particles is weakened, the results in Figs 3 and S5 indicate that the zinc-erythritol mixture weakens the internal interactions of the polysaccharides.

The use of dried polysaccharides in the AFM experiment is an important limitation of this assay that should be noted. Since natural oral biofilms are not completely dry, the dried polysaccharides used in this study may be different from their natural state. For more reliable results, it may be desirable to use wet polysaccharides. However, because wet polysaccharides are swollen with water and forces such as viscoelasticity become prominent, it is very difficult to reproducibly measure the properties of polysaccharides in practice. For this reason, we decided to evaluate the physical properties of dried polysaccharides. Although dried polysaccharides do not exactly mimic their natural state, the observation that exopolysaccharides isolated from an actual *S. mutans* biofilm are affected by the zinc chloride-erythritol mixture would be still significant.

Intra- and inter-molecular hydrogen bonds are some of the dominant forces involved in the aggregation of exopolysaccharides in biofilms^34^. To penetrate into biofilms, these abundant hydrogen bonds need to be weakened. The hydroxyl groups of erythritol may interfere with the hydrogen bonding between hydroxyl groups of polysaccharides, and an activity only occurs when erythritol is used at high concentrations^27,28^. However, in this study, the use of a much lower concentration of erythritol (19.8 mM, 0.24% by weight) caused this effect. We speculate that this activity may be triggered by the formation of cationic complexes composed of erythritol and zinc chloride. According to our hypothesis, the cationic complexes may have been transferred into biofilms containing anionic components, such as bacterial lipids, by electrostatic interactions. It is also presumed that zinc chloride and erythritol may accumulate in biofilms and efficiently act to weaken the cohesion of exopolysaccharides, allowing zinc chloride to penetrate biofilms and have an antimicrobial effect, even against mature biofilms. This progression of events is a hypothetical mechanism by which zinc-erythritol complexes kill bacteria embedded in mature biofilms.

This hypothetical mechanism was further validated by demonstrating that the diffusion efficiency of the zinc chloride-erythritol mixture is superior to that of zinc chloride itself. We conducted a disk diffusion test to measure the zone of drug diffusion in agar plates (Fig. S9)^35^. While the diffusion efficiency of zinc chloride itself was poor, it is clearly shown that the presence of erythritol broadened the diffusion zone although the expansion was not dramatic. Although diffusion within an agar plate is not the same as diffusion into biofilms, it is indirect evidence supporting our hypothetical mechanism.

In this study, we used *S. mutans* to establish a biofilm model as it is the most representative oral microorganism that cause dental caries^36^. However, the actual oral biofilm is not composed of a single bacterial species, but rather is formed by the complex interactions of various microorganisms. Although the zinc chloride-erythritol mixture assayed in this study exhibits bactericidal activity against *S. mutans* biofilms, the effect may be weak against a real oral biofilm. Therefore, further studies will be required to determine whether the zinc chloride-erythritol mixture has an effect in a well-established microcosm biofilm model or on actual dental plaques as a clinical study.

The zinc-erythritol complexes had bactericidal activity against biofilms, but did not directly remove biofilm. This can be seen in the CLSM results (Fig. 2). After the treatment with zinc-erythritol complexes, microorganisms inside the biofilm were killed, but the thickness of the biofilm did not decrease. Extracellular DNA and dead cells are components of biofilms and may also play a role in supporting the growth of surviving bacteria. However, considering previous clinical reports on the anti-calculus effect of zinc chloride^29,30^, we expect that our research will contribute to improving its efficacy, since its antibiofilm activity is dramatically enhanced by erythritol. In addition, further studies assessing the effectiveness of using zinc-erythritol complexes with substances that directly remove the mass of biofilms would allow our findings to be better utilized for oral hygiene.

Due to the close relationship between cytotoxicity and antimicrobial activity, bacteria in mature biofilms were not significantly eradicated at concentrations at which no cytotoxicity was observed. In particular, 6.6 mM zinc chloride was also cytotoxic to human oral keratinocytes. Because of the intrinsic toxicity of zinc chloride, its indiscriminate use should be avoided. However, despite its toxicity, it may not seriously threat to human health when used for a short time (<3 min) as an oral care product, such as in toothpastes and mouthrinses. Note that the results shown in Fig. 4 show the viability of cells contacted with zinc chloride for 24 h. Actually, zinc chloride and the zinc chloride-erythritol mixture were shown to exhibit no cytotoxicity to human oral keratinocytes when contacted for 3 min (Fig. S10). In addition, clinical trials of oral care products with 6.6 mM zinc chloride have reported no toxic side effects^29,37^.

The result that the toxicity of zinc chloride did not increase after the formation of complexes with erythritol has significant practical applications. Zinc chloride is widely used in oral health care for a variety of purposes, such as in the treatment of halitosis, the management of gingivitis, and the inhibition of calculus formation^29,38–40^. These oral health benefits from the use of zinc chloride are based on its ability to control plaque by killing microorganisms. In this case, there is an inevitable trade-off between efficacy and toxicity. However, the results of this study identified a means of dramatically improving the efficacy of zinc chloride without increasing its toxicity. This was made possible by weakening the internal interactions between exopolysaccharides in biofilms, which are an obstacle to the action of zinc chloride, without directly improving bactericidal activity. This approach allowed us to avoid toxicological issues that would typically be concomitant with an increase in efficacy. Another important point is that because no chemical synthesis process is required, no additional toxicological problems can arise from by-products and residual reactants. Therefore, the results in this study can be applied to humans without concern for unpredictable toxicity. In conclusion, the described method for effectively removing mature biofilms using zinc chloride and erythritol may have great potential to be extensively used in a variety of practical applications.

## Methods

### Preparation and analysis of zinc-erythritol complexes

Zinc chloride (Sigma-Aldrich, USA), erythritol (Ingredion Korea, Korea), glycerol, xylitol, and sorbitol (all from Daejung Chemicals, Korea) were dissolved at specific ratios in distilled water (DW) for use in the described experiments. To optimize the molar ratio of zinc chloride and erythritol, samples with different ratios were prepared. The concentration of zinc chloride was fixed at 6.6 mM, and erythritol was added at specific ratios. For the experiment shown in Fig. 1b, aqueous solutions containing ZnCl_2_-glycerol, -erythritol, -xylitol, and -sorbitol were prepared. The molar ratio of ZnCl_2_-glycerol and ZnCl_2_-erythritol was 1:3 and that of ZnCl_2_-xylitol and ZnCl_2_-sorbitol was 1:4.

### Formation of *S. mutans* biofilms

*S. mutans* ATCC 25175 was purchased from the Korean Collection for Type Cultures (KCTC). The bacteria were pre-cultured in brain-heart infusion (BHI; Difco, USA) medium at 37 °C under anaerobic conditions until reaching an OD_600_ of 1.0. The cultured bacteria were then diluted 1:100 in fresh BHI medium containing 1% (w/w) sucrose (Daejung Chemicals). The diluted solution was transferred to 96-well plates (Nunc, Germany), and the bacteria were further incubated at 37 °C under anaerobic conditions for 24 h to form biofilms.

### Assessment of bactericidal activity

Bactericidal activity against *S. mutans* biofilms was assessed via the Alamar blue assay in microplates^41^. After culturing *S. mutans* for 24 h to form biofilms, suspended bacteria were removed by a pipette. Next,100 μL of sample solutions were gently added to wells containing the biofilms without washing. The treated solutions were removed using a pipette after 10 min. Then, DW containing 10% (v/v) Alamar blue dye (Thermo Fisher Scientific, USA) was added to the wells. If there was no washing step before the Alamar blue assay, when 660 mM ZnCl_2_ was used for the experiments in Fig. 1b, residual ZnCl_2_ had a significant effect on the results of the assay. Therefore, in the experiments using 660 mM ZnCl_2_, a washing step was performed. However, when zinc chloride was used at concentrations lower than 66 mM, the absence of the washing step had no effect on the results. Therefore, the washing step was optional. When a washing step was added, no matter how carefully it was performed, we could not avoid a slight loss of biofilm due to application of shear force, resulting in poor reproducibility. Thus, we skipped the washing step before the Alamar blue assay. After incubation with Alamar blue dye for approximately 30 min, the fluorescence intensity at 590 nm was measured using a Wallac Victor3 1420 instrument (PerkinElmer, USA) to determine the amount of viable bacteria. In these experiments, the control group was treated with DW. The bactericidal efficiency in the test groups treated with zinc chloride, erythritol or the zinc chloride-erythritol mixture was calculated based on the fluorescence intensity obtained from the control group. All reported data were obtained by repeating experiments at least five times. In Fig. 1a, p-values was calculated using a Student’s t-test (*p < 0.05, **p < 0.01, ***p < 0.001, ****p < 0.0001).

### Analysis by CLSM

*S. mutans* cells were cultured in an IBIDI μ-slide 8-well plate (Ibidi, Germany) for 24 h at 37 °C under anaerobic conditions in BHI medium containing 1% (w/w) sucrose. Bacterial membrane integrity was evaluated using a BacLight live/dead bacterial viability kit (L-7012, Molecular Probes, USA)^42^. This kit contains SYTO9^TM^ and PI to distinguish between live and damaged cells, respectively. A BacLight staining solution was prepared by mixing 3 μL of dye and 1 mL of DW. The prepared *S. mutans* biofilm was treated with sample solutions containing 6.6 mM zinc chloride and 19.8 mM erythritol for 10 min, and the sample solutions were removed. Next, the prepared BacLight staining solution was added to the wells according to the manufacturer’s protocol, and the plate was incubated for 15 min in the dark. After washing, the stained biofilms were imaged in DW using an LD C-Apochromat 40×/1.2 NA water objective lens and an LSM 710 (Carl Zeiss, Germany) as z-stacks. We calculated the number of green and red pixels in each image and reconstructed three-dimensional images using ImageJ (NIH, http://rsb.info.nih.gov/ij/). In this experiment, the control group was a biofilm treated with DW, while the test groups were biofilms treated with zinc chloride, erythritol or the zinc chloride-erythritol mixture.

### Determination of hardness of exopolysaccharide particles isolated from biofilms

An *S. mutans* biofilm was prepared in BHI medium with 1% (w/w) sucrose for 24 h. After the formation of biofilm, suspended bacteria and medium were removed, and the established biofilm was washed with DW. Next, 0.5 N sodium hydroxide (NaOH) was added to the biofilm. By the treatment with NaOH, the biofilm was disrupted. The solution containing the disrupted biofilm and NaOH was centrifuged at 10,000 × g for 30 min. The supernatant was collected and neutralized with hydrochloric acid (HCl). After neutralization, the solution was stored at 4 °C for 48 h and was subsequently centrifuged at 10,000 × g for 30 min to separate the extracellular materials of the biofilm from lysed bacterial debris. The pellet fraction, which primarily consisted of water-insoluble polysaccharides, was obtained after washing five times with DW. Subsequently, a polysaccharide powder was obtained through lyophilization. The obtained polysaccharides were dispersed at a concentration of 0.1% (w/w) in solutions containing 6.6 mM zinc chloride, 19.8 mM erythritol and mixtures thereof. The polysaccharide suspensions were incubated at room temperature for 10 min and then were separated by filtration through polycarbonate membranes with a pore size of 400 nm (Avanti Mini-Extruder, Avanti polar lipids, USA). The polysaccharides were washed by passing DW through the membrane, and membranes containing the treated polysaccharides were dried at room temperature and used for analysis by AFM.

AFM measurements were performed using an XE-100 (Parksystems, Korea). Topographic images and force-distance curves were obtained using an NSC-36C cantilever with a spring constant of k = 0.6 N m^−1^ (Mikromasch, Germany). We scanned topography after checking a set-point value to confirm that the scan was non-destructive to polysaccharide particles. The deflection of cantilevers that occurred when the tip moved downward into polysaccharide particles was monitored in a force-distance curve. In this experiment, the control group was polysaccharide particles contacted with DW, and the test group was polysaccharide particles contacted with zinc chloride, erythritol or the zinc chloride-erythritol mixture.

### Measurement of cell viability

Human oral keratinocyte cells (#2610, ScienCell Research Laboratories, USA) were cultured in Oral Keratinocyte Medium (OKM; ScienCell Research Laboratories). The cells were seeded in 96-well plates (5,000 cells well^−1^) and incubated at 37 °C in 5% CO_2_ for 24 h. Zinc chloride and erythritol were added to wells containing cells and OKM, and the cells were further incubated for 24 h. After incubation, the culture medium was replaced with a fresh medium. Cell viability was determined using a Cell Counting Kit-8 (CCK-8; Dojindo, Japan) according to the manufacturer’s protocol. Ten microliters of CCK-8 solution was added to each well of the plate, which was then incubated for 1 h. Cell viability was determined by scanning at 450 nm. The negative and positive control groups were water-treated cells and cell-free wells, respectively. The percentages of viable cells were calculated based on values from the negative and positive controls. All reported data were obtained by repeating experiments 10 times.

## Conclusions

The results of this study demonstrate a coordination compound that can effectively kill microorganisms embedded in mature *S. mutans* biofilms. Zinc chloride and erythritol formed cationic complexes in water, and the optimal molar ratio of zinc chloride/erythritol was determined to be 1:3. The cationic complexes weakened intra- and inter-molecular interactions of exopolysaccharides in biofilms. The exopolysaccharides that lost their cohesive forces allowed the penetration of the complexes. The complexes that penetrated into biofilms were effective at killing bacteria. Furthermore, the formation of the complexes caused no increase in the intrinsic cytotoxicity of zinc chloride. This simple method of effectively eradicating bacteria embedded in mature biofilms may have great potential to be extensively used in a variety of practical applications.

## Electronic supplementary material

Supplementary Information
