# Supplementary material for: Penetration of an antimicrobial zinc-sugar alcohol complex into Streptococcus mutans biofilms
Source: Sci Rep. 2018 Nov 1;8:16154. doi: 10.1038/s41598-018-34366-y (PMC6212478; doi:10.1038/s41598-018-34366-y)
Supplement: Supplementary file 1 — Supplementary Information [file 41598_2018_34366_MOESM1_ESM.docx]

Supplementary Information

Penetration of an antimicrobial zinc-sugar alcohol complex into *Streptococcus mutans* biofilms

Jong Hyun Lim*, Yongbeom Jeong, Sang-Hun Song, Jae Hyun Ahn, Jeong Rae Lee
and Sang-Min Lee

Magok R&D Center, LG Household & Health Care, Gangseo-gu, Seoul 07795, Republic of Korea

E-mail: jonghyun16@lgcare.com


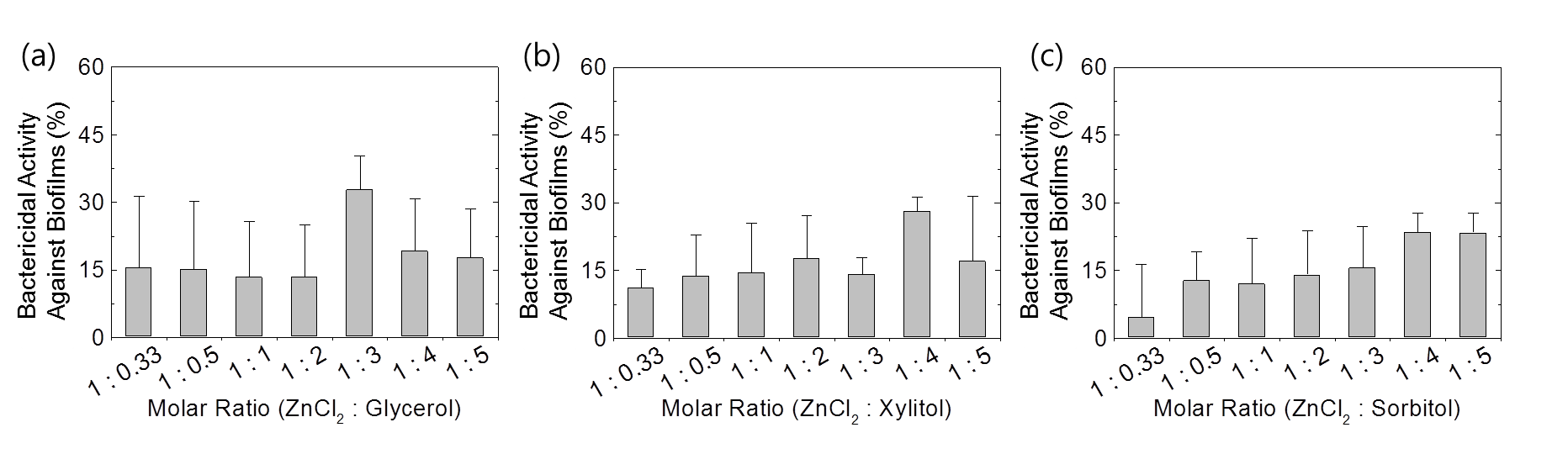


**Figure S1.** Bactericidal activity of (**a**) ZnCl_2_-glycerol, (**b**) ZnCl_2_-xylitol and (**c**) ZnCl_2_-sorbitol against *S. mutans* biofilms.

The concentration of ZnCl_2_ used was fixed (6.6 mM), and sugar alcohols were added at the indicated ratios. The biofilms were immersed for 10 min, and the bactericidal activity against the biofilm was measured using the Alamar blue assay described in the Materials and Methods. Each value and error bar indicates the mean and standard deviation (SD), respectively (*n* = 5).


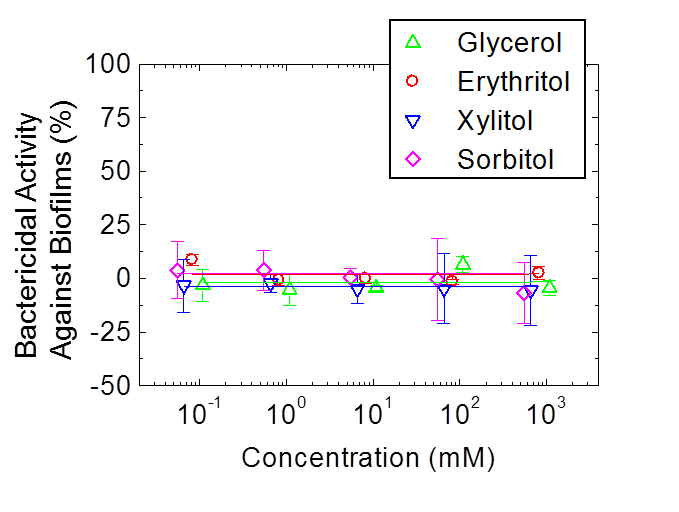


**Figure S2.** Bactericidal activity of glycerol, erythritol, xylitol and sorbitol against *S. mutans* biofilms. The sugar alcohols used did not affect the mature biofilm.

The biofilms were immersed for 10 min, and the bactericidal activity against the biofilms was measured using the Alamar blue assay described in the Materials and Methods. Each value and error bar indicates mean and SD, respectively (*n* = 5-10).


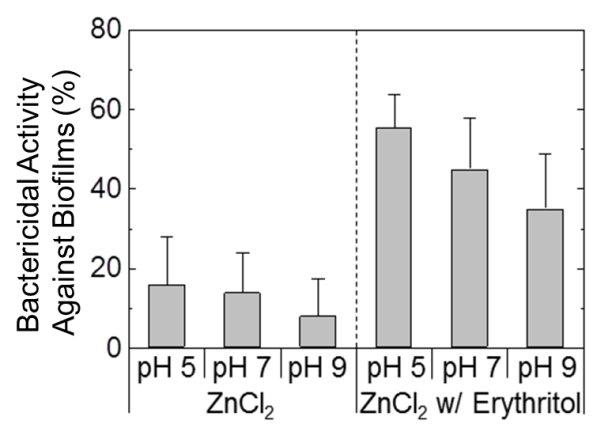


**Figure S3.** Effect of pH conditions on the antimicrobial activity against *S. mutans* biofilms. An increase in pH resulted in a decrease in antibacterial activity of zinc chloride and zinc-erythritol complexes.

The concentrations of ZnCl_2_ and erythritol used were 6.6 and 19.8 mM, respectively. The pH of solutions containing zinc chloride and the zinc-erythritol mixture was adjusted to 5, 7 or 9 with HCl and NaOH. The selected pH values of tested solutions are the same as those of typical oral care products currently on the market. The biofilms were immersed in each solution for 10 min, and the bactericidal activity against the biofilms was measured using the Alamar blue assay described in the Materials and Methods. Each value and error bar indicates the mean and SD, respectively (*n* = 5).

**
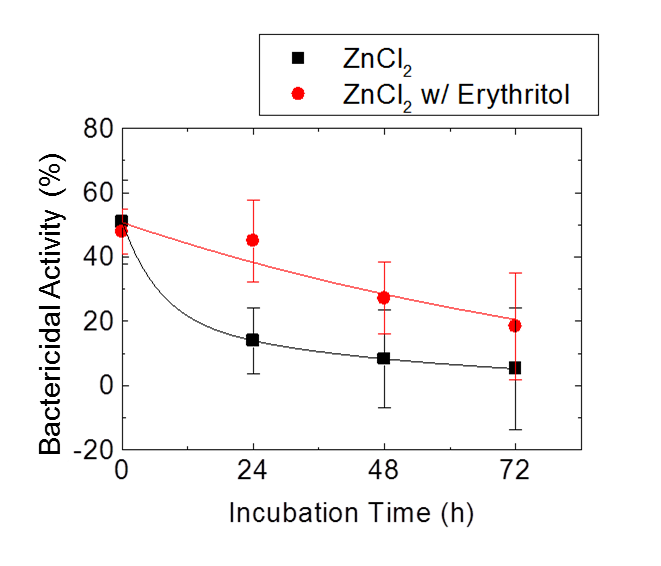
**

**Figure S4.** Effect of biofilm maturity on antibiofilm activity of zinc-erythritol complexes. At a time of 0 h, the biofilms had not yet formed. The biofilms were immersed in a solution containing 6.6 mM zinc chloride and 19.8 mM erythritol for 10 min. Each value and error bar indicates the mean and SD, respectively (*n* = 5-10).

Antimicrobial activity on planktonic bacteria prior to the formation of biofilms (i.e., when the incubation time was 0 h) was evaluated using a colony-forming units (CFU) count method. *S. mutans* cells were cultured in brain-heart infusion (BHI) medium until OD_600_ = 0.5, at which time the *S. mutans* cells were in the exponential growth phase. 100 μL of the bacterial solution and 100 μL of the sample solution containing zinc chloride or the zinc chloride-erythritol mixture were mixed. In this experiment, the concentrations of zinc chloride and erythritol in the sample solutions were 12.4 and 39.6 mM, respectively. Because the bacterial solution and the sample solutions were mixed at a ratio of 1:1, the final concentrations of zinc chloride and erythritol during contact were 6.6 and 19.8 mM, respectively. The mixed solutions were incubated at room temperature for 10 min. After incubation, the bacterial cells were immediately separated from the sample solutions by centrifugation at 12,000 × g for 5 min. The supernatants were removed, and the pellets were resuspended using 1 mL of fresh BHI medium. The resuspended solutions were diluted 10^-4^ times with BHI medium. Next, 10 μL of the diluted solutions were spread onto agar plates. The plates were incubated overnight at 37°C under anaerobic conditions, and the number of colonies was counted. The percentage of bactericidal activity was calculated based on the number of colonies from bacterial samples treated with distilled water (DW).

The mature biofilms were prepared as described in the Materials and Methods. For biofilms cultured for more than 24 h, culture medium was replaced every 24 h. The Alamar blue assay described in the Materials and Methods was used to determine the bactericidal activity against biofilms.


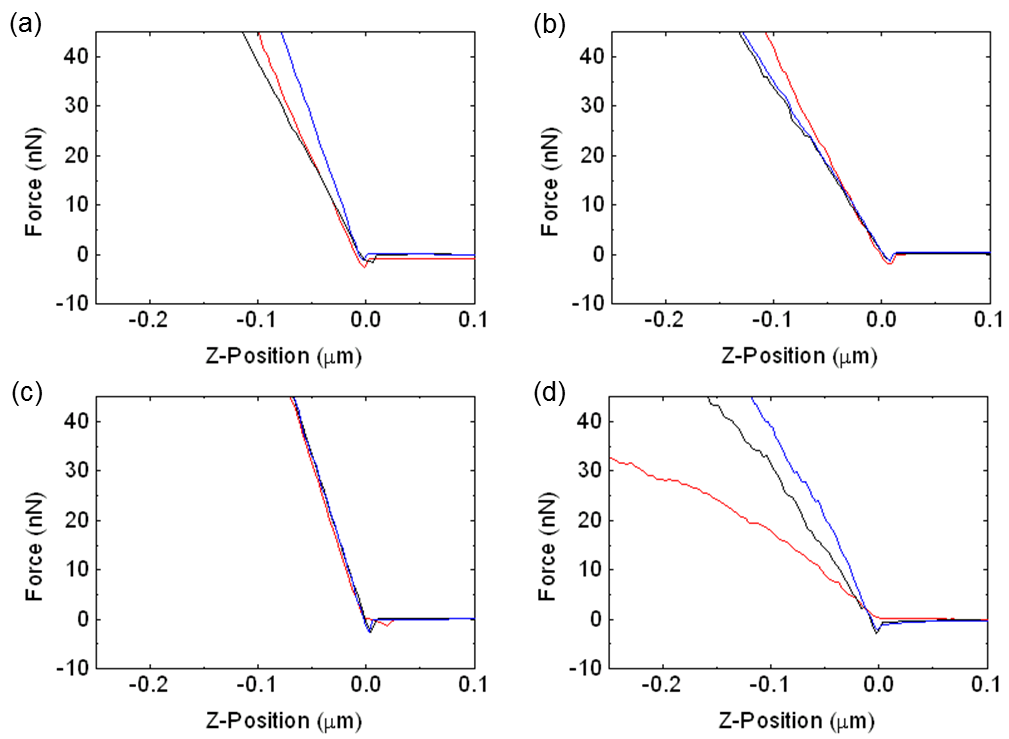


**Figure S5.** Force-distance curves obtained by moving the AFM tip downward into polysaccharide particles treated with (**a**) DW, (**b**) zinc chloride, (**c**) erythritol and (**d**) a zinc-erythritol mixture for 10 min.

The polysaccharide particles were treated with 6.6 mM zinc chloride, 19.8 mM erythritol and mixtures thereof. The data sets plotted onto one graph were independently measured on different polysaccharide particles. The slope of the force-distance curve obtained from polysaccharide particles treated with zinc-erythritol complexes decreased with the penetration of the AFM tip in three independent measurements.

**Figure S6.** Viability of human oral keratinocytes after treatment with erythritol. Erythritol showed no cytotoxicity at concentrations below 10^-2^ M. Each value and error bar indicates the mean and SD, respectively (*n* = 10).


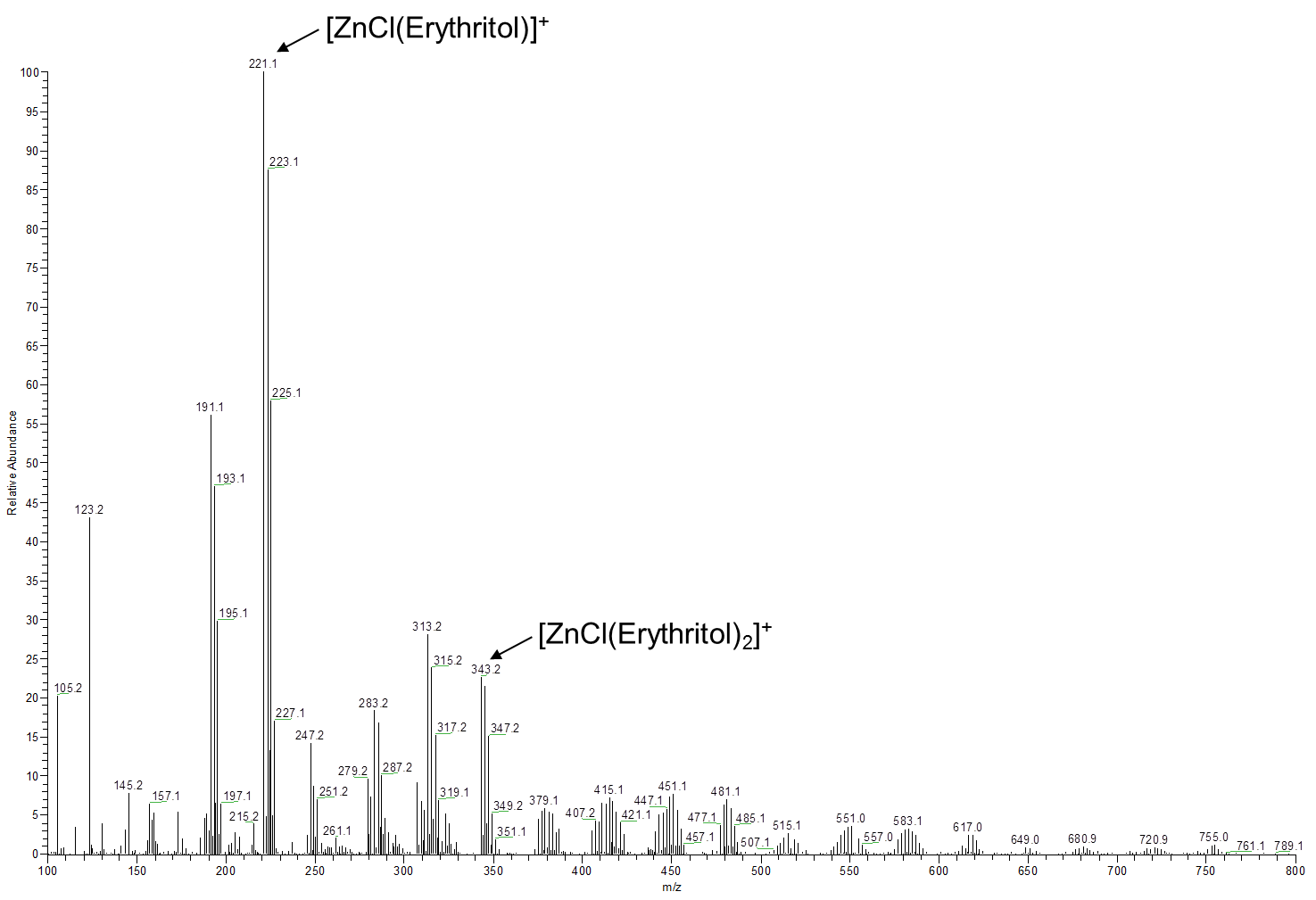


**Figure S7.** Fast atom bombardment mass (FAB-MS) spectrum of a mixture of zinc chloride and erythritol with a molar ratio of 1:3. The mass of the zinc-erythritol mixture was analyzed after its dissolution in deionized water. Split peaks appeared due to isotopes of zinc and chlorine. The presence of [ZnCl(erythritol)]^+^ and [ZnCl(erythritol)_2_]^+^ was confirmed in the mass spectrum.

Mass analysis was conducted by fast atom bombardment (FAB) using a Thermo DFS high-resolution magnetic sector mass spectrometer (Thermo Scientific, Germany). For the FAB-MS analysis, zinc chloride and erythritol were first mixed at a molar ratio of 1:3 and then was dissolved at a concentration of approximately 1% (w/w) in DW. Electron energy, emission current and acceleration voltage were set at -20 eV, 0.01 mA and 5 kV, respectively.


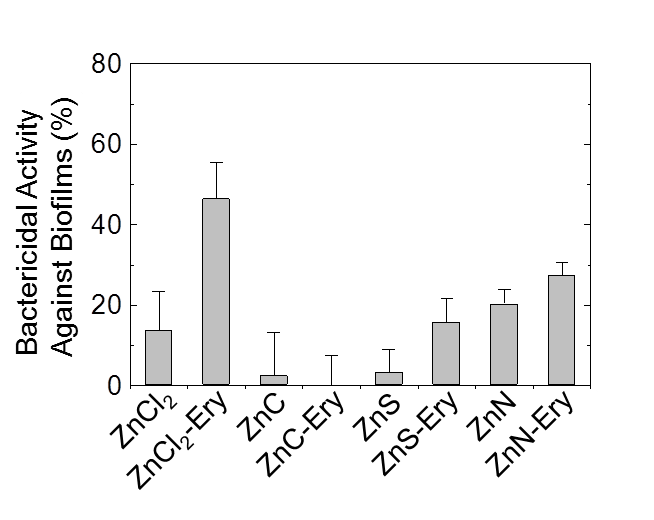


**Figure S8.** Bactericidal activity of zinc salts and zinc salt-erythritol mixtures against *S. mutans* biofilms. Ery: erythritol; ZnC: zinc citrate; ZnS: zinc sulfate; and ZnN: zinc nitrate.

The concentrations of zinc salts and erythritol used were 6.6 and 19.8 mM, respectively, at molar ratios of 1:3. The biofilms were immersed in each solution for 10 min, and the bactericidal activity against biofilms was measured using the Alamar blue assay described in the Materials and Methods. Each value and error bar indicates the mean and SD, respectively (*n* = 5).


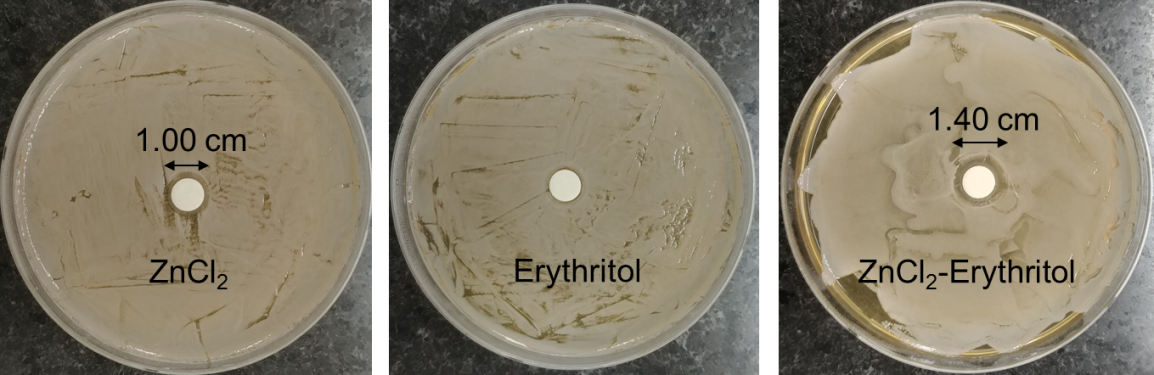


**Figure S9.** Disk diffusion tests showing zones in which zinc chloride, erythritol and a zinc chloride-erythritol mixture inhibited the growth of *S. mutans* on agar medium.

The disk diffusion assay was performed by inoculating 100 μL of a *S. mutans* solution at a concentration of approximately 1 × 10^7^ CFU mL^-1^ onto the surface of brain-heart infusion (BHI) agar plates. Paper disks (diameter 8 mm; Advantec, Japan) were placed on the agar plates just after the inoculation, and 50 μL of sample solutions were spotted onto the paper disks. The sample solutions contained zinc chloride, erythritol or a zinc chloride-erythritol mixture. The concentrations of zinc chloride and erythritol used were 66 and 198 mM, respectively. The plates were incubated for 16 h at 37°C under anaerobic conditions. After the incubation, the zone of growth inhibition around each disk was determined. The diameter of the zone is generally related to the susceptibility of the inoculated microorganism and to the diffusion rate of the tested drug through the agar medium. In this experiment, because the same microorganism was inoculated onto each plate, the diameter of the inhibition zone was only affected by the diffusion of the tested drug.


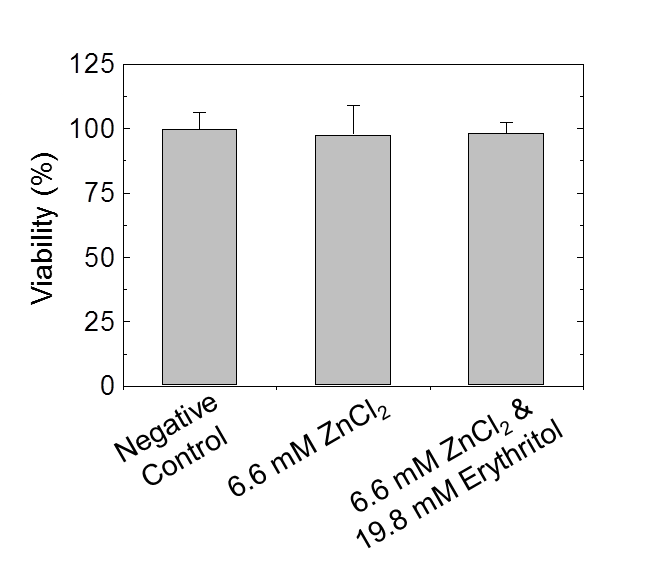


**Figure S10.** Viability of human oral keratinocytes after treatment with ZnCl_2_ and a ZnCl_2_-erythritol mixture for 3 min. The test samples showed no cytotoxicity after contact with the antibacterial agents for 3 min. Each value and error bar indicates the mean and SD, respectively (*n* = 10).
